# A nomogram integrating the clinical and CT imaging characteristics for assessing spread through air spaces in clinical stage IA lung adenocarcinoma

**DOI:** 10.3389/fimmu.2025.1519766

**Published:** 2025-04-11

**Authors:** Yantao Yang, Li Li, Huilian Hu, Chen Zhou, Qiubo Huang, Jie Zhao, Yaowu Duan, Wangcai Li, Jia Luo, Jiezhi Jiang, Zhenghong Yang, Guangqiang Zhao, Yunchao Huang, Lianhua Ye

**Affiliations:** ^1^ Department of Thoracic and Cardiovascular Surgery, Peking University Cancer Hospital Yunnan, Yunnan Cancer Hospital, The Third Affiliated Hospital of Kunming Medical University, Kunming, China; ^2^ Cancer Biotherapy Center, Peking University Cancer Hospital Yunnan, Yunnan Cancer Hospital, The Third Affiliated Hospital of Kunming Medical University, Kunming, China; ^3^ Department of Oncology, Qujing City Hospital of Traditional Chinese Medicine, Qujing, China; ^4^ Department of Pathology, Peking University Cancer Hospital Yunnan, Yunnan Cancer Hospital, The Third Affiliated Hospital of Kunming Medical University, Kunming, China; ^5^ Department of Radiology, Peking University Cancer Hospital Yunnan, Yunnan Cancer Hospital, The Third Affiliated Hospital of Kunming Medical University, Kunming, China

**Keywords:** clinical feature, radiologic characteristic, lung adenocarcinoma, STAS, nomogram

## Abstract

**Purpose:**

This study aimed to create a nomogram model to predict the spread through air spaces (STAS) in patients diagnosed with stage IA lung adenocarcinoma, utilizing a substantial sample size alongside a blend of clinical and imaging features. This model serves as a valuable reference for the preoperative planning process in these patients.

**Materials and methods:**

A total of 1244 individuals were included in the study. Individuals who received surgical intervention between January 2022 and May 2023 were categorized into a training cohort (n=950), whereas those treated from June 2023 to October 2023 were placed in a validation cohort (n=294). Data from clinical assessments and CT imaging were gathered from all participants. In the training cohort, analyses employing both multivariate and univariate logistic regression were performed to discern significant clinical and CT characteristics. The identified features were subsequently employed to develop a nomogram prediction model. The evaluation of the model’s discrimination, calibration, and clinical utility was conducted in both cohorts.

**Results:**

In the training cohort, multivariate logistic regression analysis revealed several independent risk factors associated with invasive adenocarcinoma: maximum diameter (OR=2.459, 95%CI: 1.833-3.298), nodule type (OR=4.024, 95%CI: 2.909-5.567), pleura traction sign (OR=2.031, 95%CI: 1.394-2.961), vascular convergence sign (OR=3.700, 95%CI: 1.668-8.210), and CEA (OR=1.942, 95%CI: 1.302-2.899). A nomogram model was constructed utilizing these factors to forecast the occurrence of STAS in stage IA lung adenocarcinoma. The Area Under the Curve (AUC) measured 0.835 (95% CI: 0.808–0.862) in the training cohort and 0.830 (95% CI: 0.782–0.878) in the validation cohort. The internal validation conducted through the bootstrap method yielded an AUC of 0.846 (95% CI: 0.818-0.881), demonstrating a robust capacity for discrimination. The Hosmer–Lemeshow goodness-of-fit test confirmed a satisfactory model fit in both groups (P > 0.05). Additionally, the calibration curve and decision analysis curve demonstrated high calibration and clinical applicability of the model in both cohorts.

**Conclusion:**

By integrating clinical and CT imaging characteristics, a nomogram model was developed to predict the occurrence of STAS, demonstrating robust predictive performance and providing valuable support for decision-making in patients with stage IA lung adenocarcinoma.

## Introduction

The Global Cancer Report 2020 highlights lung cancer as the leading cause of cancer-related mortality worldwide, with adenocarcinoma recognized as the predominant histological subtype (1). In 2021, the World Health Organization (WHO) updated the classification of lung adenocarcinomas, dividing them into *in-situ*, microinvasive, and invasive categories, based on the extent of their invasive progression (2).

The widespread adoption of early lung cancer screening programs has led to increased detection of stage IA lung adenocarcinomas, which typically present as lung nodules. Surgical excision is the main treatment modality for these patients (3). For early-stage IA lung adenocarcinoma, sublobectomy is now the treatment of choice (4–6). Nonetheless, in some instances, recurrences and metastases occur, with STAS significantly contributing to these outcomes (7).

STAS was introduced by the WHO in 2015 as a distinct pattern of invasion in invasive lung adenocarcinoma (IAC). This pattern is marked by the presence of pathological micropapillary clusters, solid nests, or single cells that extend beyond the tumor margin, infiltrating the adjacent lung parenchyma (8). Research indicates that STAS correlates with a worse prognosis, prompting several scholars to advocate for lobectomy in cases of IA lung adenocarcinoma showing STAS positivity (9–12).

Limitations exist with intraoperative frozen sections for STAS prediction, making preoperative clinical and imaging evaluations more predictive (13). Historical data suggest variability in the predictive reliability of clinical and imaging features for STAS (8, 14–16). Studies by Onozato et al. (15) and Shiono et al. (14) suggest that smoking increases the risk of STAS, although Uruga et al. (16) observed no such correlation. Similarly, Warth et al. (17) identified male gender as a risk factor for STAS, a finding not corroborated by Kadota (8).

Other research indicates that a tumor diameter exceeding 2 cm does not reliably predict STAS (18, 19). Yin et al. (20) observed that pulmonary nodules with partly solid features tend to develop STAS, while Toyokawa et al. (19) determined that a solid component over 50% is predictive of STAS. Key CT indicators such as solid nodules, spiculation, vacuoles, well-defined borders, lobulation, and pleural traction are frequently observed in tumors positive for STAS (20, 21). However, the focus on individual features has limited the overall predictive accuracy. Combining multiple diagnostic features has shown to enhance performance, with Ding et al. (22) achieving an AUC of 0.724 using a combination of CTR, tumor size, vacuoles, and spiculation, while Gao et al. (23) reached an AUC of 0.808 by integrating age, nodule type, and SUVmax.

Despite some improvements in diagnostic efficacy, the limitations of small sample sizes and variability in features persist, underscoring the need for further studies involving more comprehensive datasets. To address this gap, we propose a large-scale investigation to thoroughly assess the link between clinical and imaging features and STAS in stage IA lung adenocarcinoma. Our objectives include identifying independent predictors, developing and validating a predictive nomogram for STAS, and aiding in the selection of appropriate treatment strategies for patients with stage IA lung adenocarcinoma.

## Materials and methods

### Participants

**Figure 1:**
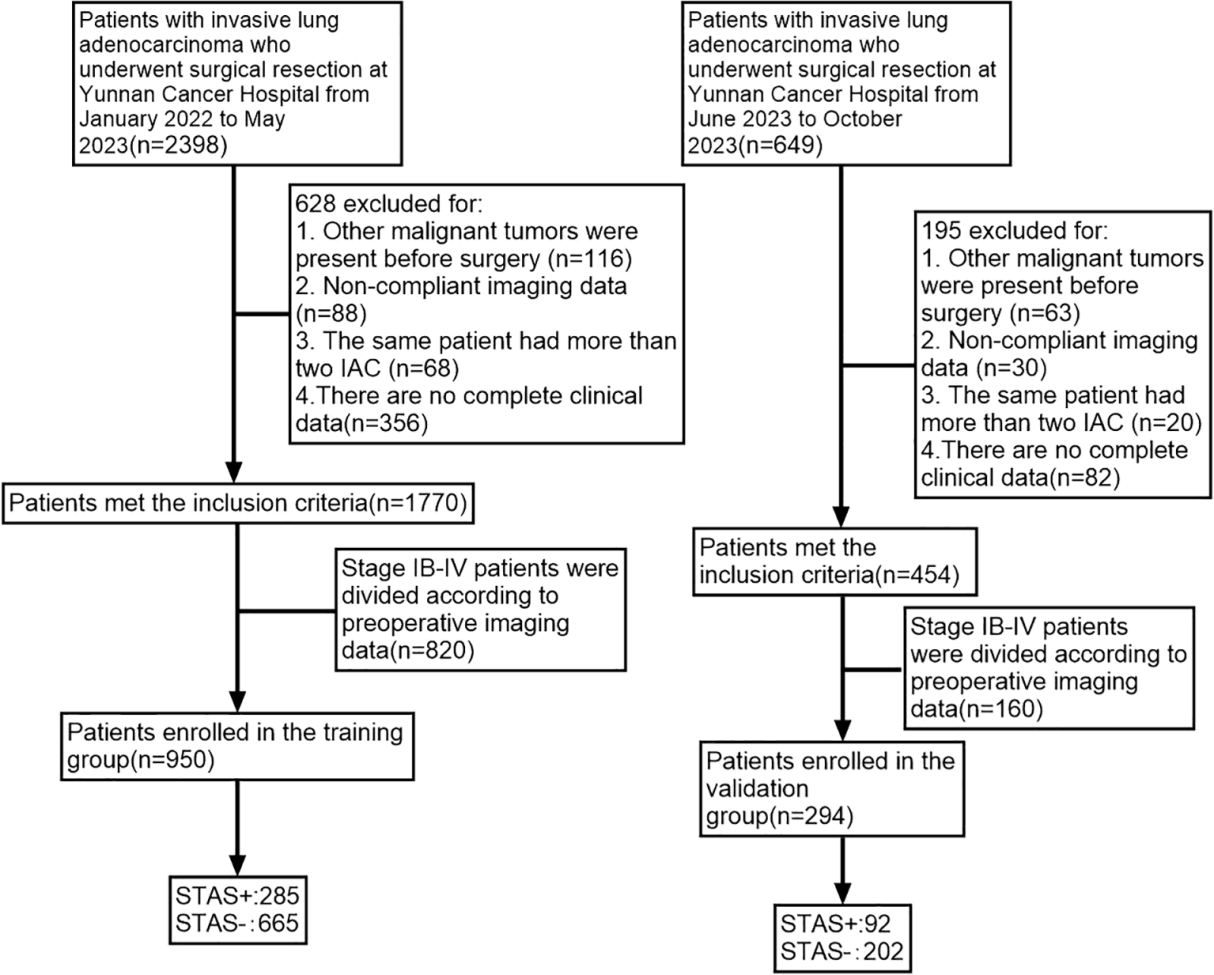
Patient screening flowchart.

Data were collected retrospectively from patients diagnosed with IAC who had surgical resection at Yunnan Cancer Hospital (Third Affiliated Hospital of Kunming Medical University) from January 2022 to October 2023. The staging process adhered to the 8th edition of the IASLC TNM staging system specifically designed for lung cancer (24, 25). Inclusion criteria included: (1) Preoperative CT reports indicating pulmonary nodules with a maximum diameter of less than 3 cm; (2) CT imaging performed at Yunnan Cancer Hospital within two weeks prior to surgery; (3) A postoperative pathological diagnosis confirming IAC; (4) Exclusion of distant metastasis through preoperative imaging techniques such as CT, PET-CT, or ultrasound; (5) Patient age of 18 years or older. Exclusion criteria were: (1) Presence of other malignant tumors prior to surgery; (2) Non-compliant imaging data; (3) Presence of more than two invasive adenocarcinoma nodules in a single patient; (4) Incomplete collection of medical, imaging, or hematology data; (5) Patients classified as stage IB-IV through preoperative imaging. Patients were allocated into two cohorts based on the date of their surgery: (1) 950 patients between January 2022 and May 2023 formed the training cohort; (2) 294 ground-glass nodules (GGNs) from June 2023 to October 2023 constituted the validation cohort (**Figure 1**).

### Ethical approval and study protocol

Under approval number KYLX2023-137, the study was given the green light by the Ethics Committee of Yunnan Cancer Hospital, which is the Third Affiliated Hospital of Kunming Medical University. The necessity to get informed consent was removed because the study was retrospective in nature. Surgical cohort, cross-sectional, and case-control studies were reported in accordance with the STROCSS criteria, and the protocol was registered with ClinicalTrials.gov (26).

### CT acquisition

Before the chest CT examination, patients participated in breathing training. Throughout the scanning procedure, subjects were placed in a supine position, with their arms raised, and were directed to take deep breaths. Following either breath-holding or tranquil respiration, a breath-holding scan was performed from the apex of the lung to its base utilizing a helical sweep methodology. The experimental conditions were established with a tube voltage of 120 kilovolt (kV) and a current of 100 milliampere (mA), alongside a pitch of 1.0 and a slice thickness of 1mm. The sweep parameters consisted of a voltage setting of 70 kV and a tube current of 50 milliampere-seconds (mAs), accompanied by a resolution matrix measuring 512×512. The settings for the lung window varied between 1200 and 1500 Hounsfield Units (HU), with a window level established at -600 to -700 HU. The mediastinal window was established within a range of 400 to 500 HU, featuring a window level of 40 to 50 HU.

### Clinical and imaging features analysis

Clinical data collected included: (1) demographic characteristics including sex, age, tobacco use history, and family history of cancer; (2) Preoperative blood markers encompassed Carcinoembryonic Antigen (CEA), Carbohydrate Antigen 125 (CA125), as well as ratios including the platelet-lymphocyte ratio (PLR) and neutrophil-lymphocyte ratio (NLR). Additionally, the systemic inflammation index (SII) was determined by the product of platelet count and NLR.

Two senior diagnostic chest radiologists, unaware of the patients’ pathological outcomes and fundamental demographic details, meticulously assessed and recorded the CT imaging characteristics. The evaluated imaging characteristics comprised: (1) Air bronchogram sign: visible as tubular air-density within bronchi at various segments on consecutive CT slices; (2) Cavitation sign: characterized by air or low-density areas within nodules measuring less than 5 mm, with features such as smooth, irregular contours or faintly defined boundaries; (3) Pleural indentation sign: tent-like or linear opacities between the pleura and lesions, or star-shaped shadows; (4) Vascular convergence sign: displacement and convergence of blood vessels towards a lesion, indicative of traction or direct association with the lesion; (5) Lobulation sign: irregular, arc-like contours on the nodule’s perimeter, possibly forming multiple lobular projections with serrated or wavy intervening notches; (6) Spiculation sign: radial, sharp projections from the nodule edge, resembling a dense brush or thin line without branching, not adhering to the pleural surface; (7) Maximum tumor diameter: measured on axial CT images; (8) Nodule type: categorized as solid, partially solid, or pure ground-glass nodules.

### Histopathological evaluation

The surgical excision of tissue samples was followed by preservation in 10% formalin, embedding in paraffin, sectioning with a microtome, and staining using Hematoxylin and Eosin (HE). The categorization of each specimen followed the 2015 WHO Classification of Lung Tumors and the criteria established by Kadota et al. (8, 27). STAS positivity was determined by the presence of isolated cancer cells or small clusters of cancer cells within alveolar spaces, distinctly separated from the primary tumor by at least one alveolar septum. Criteria for excluding STAS, as defined by Kadota et al. (8), included: (1) mechanically displaced tumor cells or irregular clusters randomly distributed or located at the margins of the section; (2) tumor cell strands detached from interstitial lung tissue or alveolar walls due to inadequate tissue preservation. In instances where distinguishing non-tumor cells proved challenging, immunohistochemistry was utilized to confirm the status of STAS. Two pathologists, each possessing over 15 years of expertise within the Pathology Department of Yunnan Cancer Hospital, performed the evaluations independently.

### Imaging feature selection

Univariate analyses were conducted to examine the clinical and imaging characteristics between the groups identified as STAS-positive and STAS-negative within the training cohorts. Variables exhibiting a P-value < 0.05 in the univariate analysis were analyzed further through multivariate logistic regression to identify significant clinical and imaging predictors of IAC. The Variance Inflation Factor (VIF) was employed to assess collinearity among the independent variables. Clinical and imaging characteristics between the training and validation cohorts were compared using Mann–Whitney U tests and chi-square tests.

### Model construction and performance assessment

Multivariate logistic regression models were employed to evaluate the influence of various factors on the construction of the nomogram. Only variables with a P-value < 0.05 in the multivariate analysis were included in the final nomogram. The nomogram for predicting STAS in clinical stage IA lung adenocarcinoma was developed using R software. Model performance was assessed in both training and validation groups, with discriminative ability measured by AUC values, calibration by calibration curves, model fit by Hosmer–Lemeshow tests, and clinical utility by decision curve analysis (DCA). Internal validation was conducted through bootstrap resampling 1,000 times.

### Statistical methods

Independent sample t-tests were employed to compare continuous variables between STAS-positive and STAS-negative cohorts, provided these variables conformed to a normal distribution. In instances where the assumptions of normality were not satisfied, Mann–Whitney U tests were employed. The continuous variables encompassed age, NLR, PLR, SII, and the maximum diameter of the tumor. The assessment involved categorical variables including gender, tumor location, smoking history, nodule type, and levels of CEA and CA125. Additionally, signs such as vacuoles, pleural traction, lobulation, air bronchogram, vascular convergence, and spiculation were evaluated using the Pearson chi-square test. Binary logistic regression was utilized for both continuous and categorical variables that exhibited significant differences (P < 0.05) in the univariate analysis. A streamlined logistic regression model was constructed employing a backward elimination approach. All statistical analyses were performed utilizing R software (version 4.2.1) and SPSS (version 26.0), employing an optimal cutoff determined by the maximum Youden’s index. A P-value below 0.05 was deemed statistically significant.

## Results

### Clinical and pathological characteristics

A total of 1,244 individuals participated in the study, comprising 451 males (36.3%) and 793 females (63.7%). In the training cohort, the STAS-positive group comprised 285 patients, while the STAS-negative group included 665 patients. The validation cohort consisted of 92 STAS-positive and 202 STAS-negative patients. No significant differences in clinical or CT characteristics were observed between the training and validation cohorts, supporting their use for model development and evaluation. Detailed clinical and CT characteristics of the patients are outlined in **Table 1**.

**Table 1:** Comparison of clinical and CT features between the training group and the validation group.

Variables	Total (N=1244)	Train group (N=950)	Validation group (N=294)	*P*
age, Median (Q1,Q3)	56.00 (50.00,63.00)	56.00 (50.00, 63.00)	57.00 (50.00, 63.00)	0.301
Maximum diameter, Median (Q1,Q3)	1.50 (1.10,2.10)	1.50 (1.10, 2.10)	1.50 (1.10, 2.00)	0.116
NLR, Median (Q1,Q3)	1.73 (1.32,2.28)	1.76 (1.32, 2.29)	1.63 (1.31, 2.22)	0.144
PLR, Median (Q1,Q3)	120.45 (96.32,155.30)	121.28 (97.40, 155.63)	118.19 (94.76, 153.38)	0.385
SII, Median (Q1,Q3)	386.81 (281.43,561.81)	394.70 (282.62, 571.40)	362.86 (280.84, 516.94)	0.113
gender, n (%)				0.292
male	451 (36.3%)	352 (37.1%)	99 (33.7%)	
female	793 (63.7%)	598 (62.9%)	195 (66.3%)	
Family history of cancer, n (%)				0.093
No	1143	866	277	
Yes	101	84	17	
Smoking history, n (%)				0.941
No	967 (77.7%)	738 (77.7%)	229 (77.9%)	
Yes	277 (22.3%)	212 (22.3%)	65 (22.1%)	
STAS, n (%)				0.673
No	867 (69.7%)	665 (70.0%)	202 (68.7%)	
Yes	377 (30.3%)	285 (30.0%)	92 (31.3%)	
Tumor location, n (%)				0.532
Superior lobe of right lung	397 (31.9%)	291 (30.6%)	106 (36.1%)	
Middle lobe of right lung	92 (7.4%)	71 (7.5%)	21 (7.1%)	
Inferior lobe of right lung	256 (20.6%)	201 (21.2%)	55 (18.7%)	
Upper lobe of left lung	296 (23.8%)	230 (24.2%)	66 (22.4%)	
Inferior lobe of left lung	203 (16.3%)	157 (16.5%)	46 (15.6%)	
Nodule type, n (%)				0.083
Ground glass nodule	157 (12.6%)	109 (11.5%)	48 (16.3%)	
Part solid nodules	592 (47.6%)	461 (48.5%)	131 (44.6%)	
Solid nodules	495 (39.8%)	380 (40.0%)	115 (39.1%)	
lobulation, n (%)				0.866
No	973 (78.2%)	742 (78.1%)	231 (78.6%)	
Yes	271 (21.8%)	208 (21.9%)	63 (21.4%)	
spiculation, n (%)				0.402
No	890 (71.5%)	674 (70.9%)	216 (73.5%)	
Yes	354 (28.5%)	276 (29.1%)	78 (26.5%)	
Vacuole sign, n (%)				0.581
No	1045 (84.0%)	795 (83.7%)	250 (85.0%)	
Yes	199 (16.0%)	155 (16.3%)	44 (15.0%)	
Pleura traction sign, n (%)				0.065
No	589 (47.3%)	436 (45.9%)	153 (52.0%)	
Yes	655 (52.7%)	514 (54.1%)	141 (48.0%)	
Air bronchogram sign, n (%)				0.965
No	1219 (98.0%)	931 (98.0%)	288 (98.0%)	
Yes	25 (2.0%)	19 (2.0%)	6 (2.0%)	
Vascular convergence sign, n (%)				0.094
No	1186 (95.3%)	911 (95.9%)	275 (93.5%)	
Yes	58 (4.7%)	39 (4.1%)	19 (6.5%)	
CEA (μg/L), n (%)				0.071
>3.4	258 (20.7%)	208 (21.9%)	50 (17.0%)	
≤3.4	986 (79.3%)	742 (78.1%)	244 (83.0%)	
CA125 (μg/L), n (%)				0.284
>35	28 (2.3%)	19 (2.0%)	9 (3.1%)	
≤35	1216 (97.7%)	931 (98.0%)	285 (96.9%)	

### Imaging features analysis and selection

Univariate analysis identified gender, smoking history, CEA, CA125, lobulation, maximum tumor diameter, spiculation, signs of vascular convergence, pleural traction, and nodule type as statistically significant variables (P < 0.05) in the training cohort (**Table 2**). Following this, binary logistic regression highlighted maximum diameter (OR=2.459, 95%CI: 1.833-3.298), nodule type (OR=4.024, 95%CI: 2.909-5.567), pleural traction sign (OR=2.031, 95%CI: 1.394-2.961), vascular convergence sign (OR=3.700, 95%CI: 1.668-8.210), and CEA (OR=1.942, 95%CI: 1.302-2.899) as independent predictors of STAS (P < 0.05) (**Table 2**). Analysis for collinearity among these five indicators showed no significant issues. Additionally, ROC curves for each risk factor were generated, and based on the Youden index, an optimal cutoff for maximum diameter was established at 1.45 cm.

**Table 2:** Multivariable Logistic Regression of clinical and CT finding and STAS of patients.

	Univariate		Multivariate	
	OR (95%CI)	*P*	OR (95%CI)	*P*
gender	0.585 (0.441-0.777)	<0.001	0.913 (0.581-1.437)	0.695
age	1.005 (0.991-1.020)	0.459		
Family cancer	0.612 (0.356-1.051)	0.075		
Smoking history	1.949 (1.418-2.679)	<0.001	1.349 (0.807-2.255)	0.253
Tumor location	1.039 (0.946-1.141)	0.424		
Maximum diameter	3.605 (2.815-4.617)	<0.001	2.459 (1.833-3.298)	<0.001
Nodule type	5.837 (4.384-7.773)	<0.001	4.024 (2.909-5.567)	<0.001
lobulation	3.018 (2.194-4.153)	<0.001	1.366 (0.925-2.017)	0.116
spiculation	3.121 (2.319-4.201)	<0.001	1.073 (0.740-1.555)	0.71
Vacuole sign	0.911 (0.623-1.333)	0.632		
Pleura traction sign	4.056 (2.964-5.550)	<0.001	2.031 (1.394-2.961)	<0.001
Air bronchogram sign	1.370 (0.534-3.517)	0.513		
Vascular convergence sign	5.035 (2.548-9.950)	<0.001	3.700 (1.668-8.210)	0.001
CEA	3.096 (2.252-4.274)	<0.001	1.942 (1.302-2.899)	0.001
CA125	3.300 (1.312-8.264)	0.011	2.309 (0.740-7.246)	0.149
NLR	1.027 (0.969-1.089)	0.371		
PLR	1.000 (0.997-1.003)	0.942		
SII	1.000 (1.000-1.000)	0.304		

### Nomogram models construction and validation

**Figure 2:**
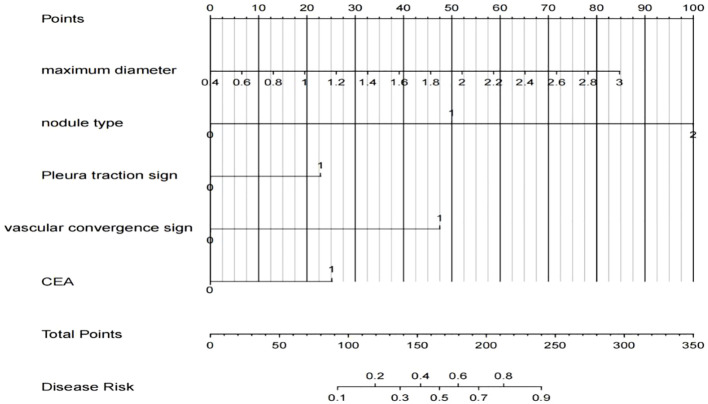
A nomogram model predicting the occurrence of STAS in IA lung adenocarcinoma patients.

**Figure 3:**
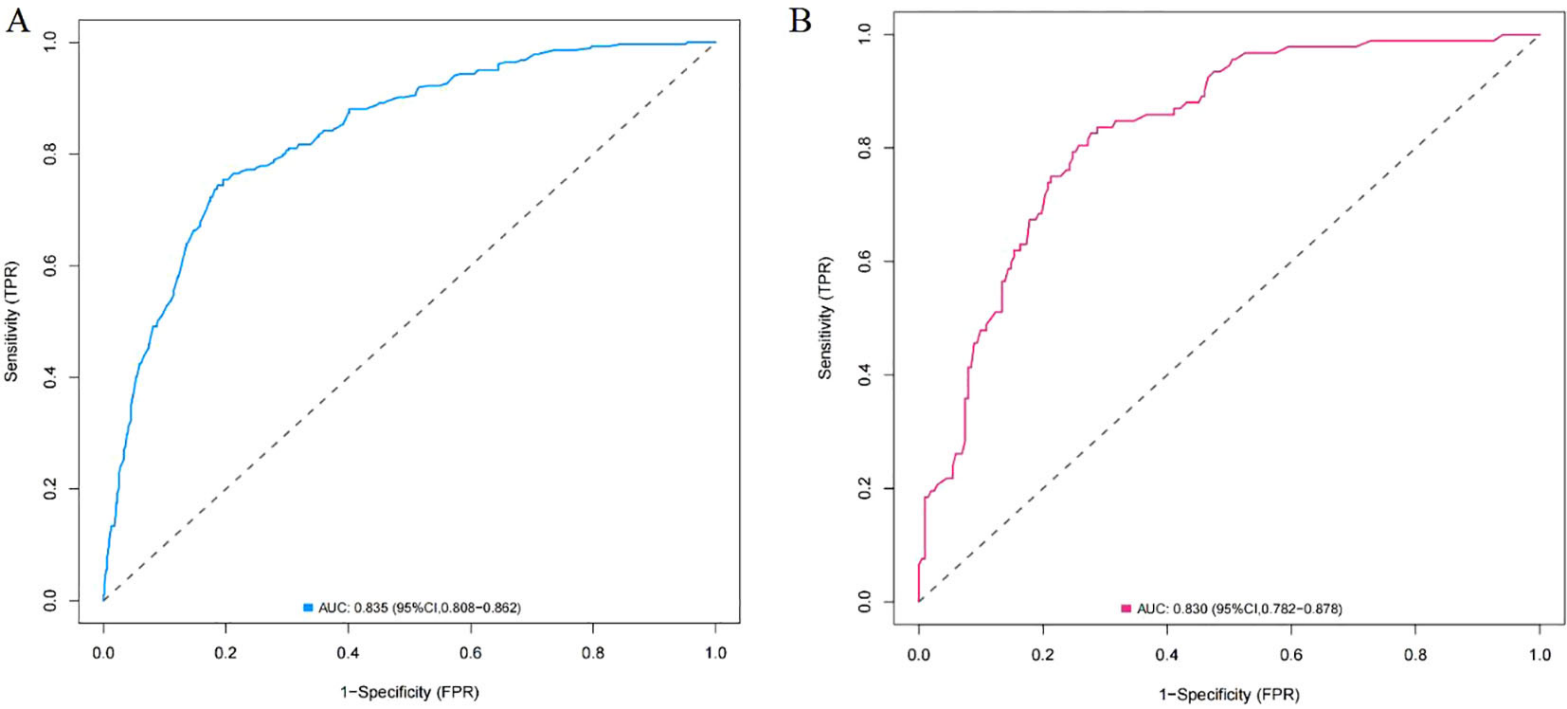
**(A)** ROC curve of the nomogram in training group. **(B)** ROC curve of the nomogram in validation group.

**Figure 4:**
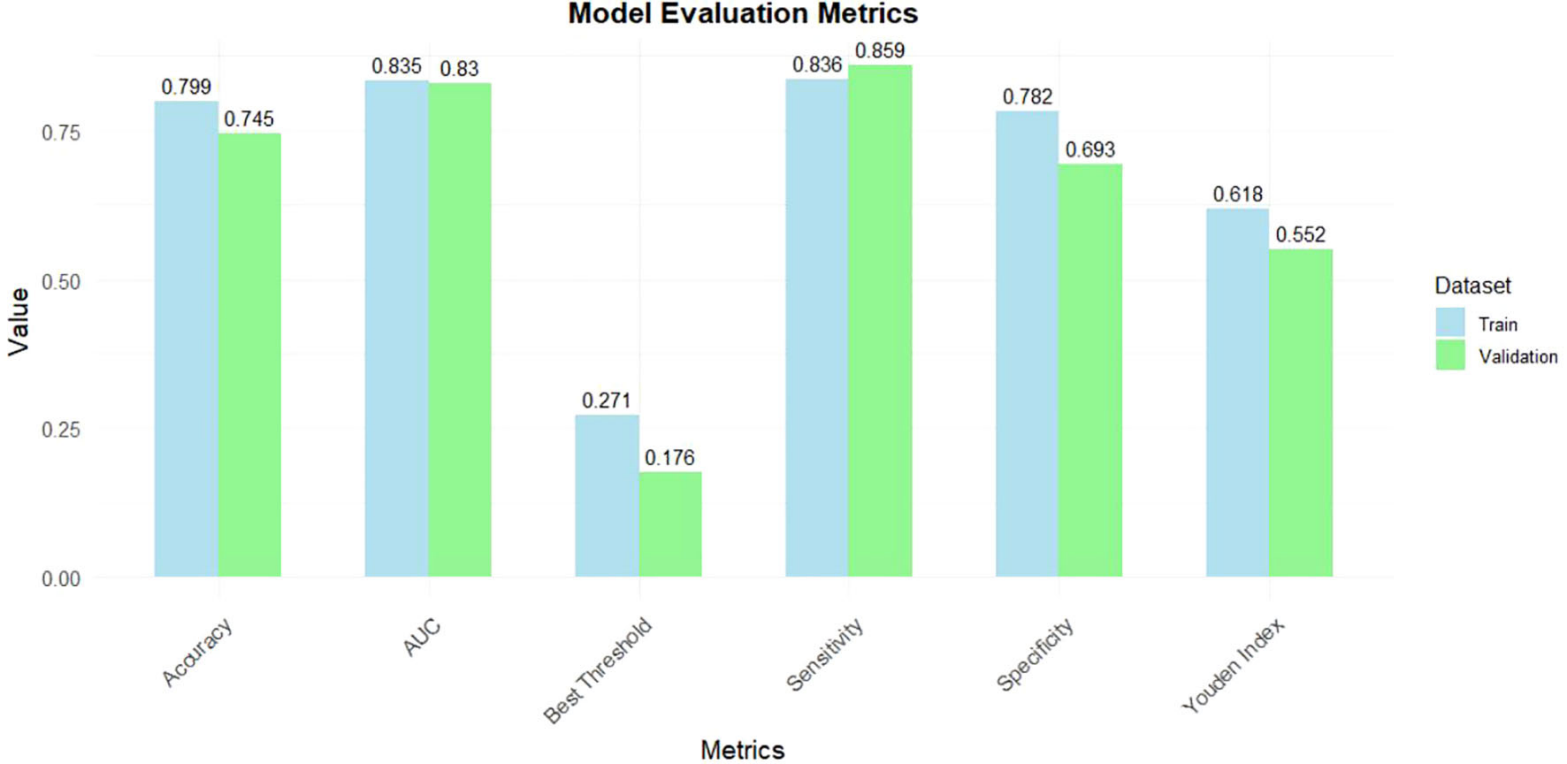
The predictive ability of the model in the training and validation groups.

**Figure 5:**
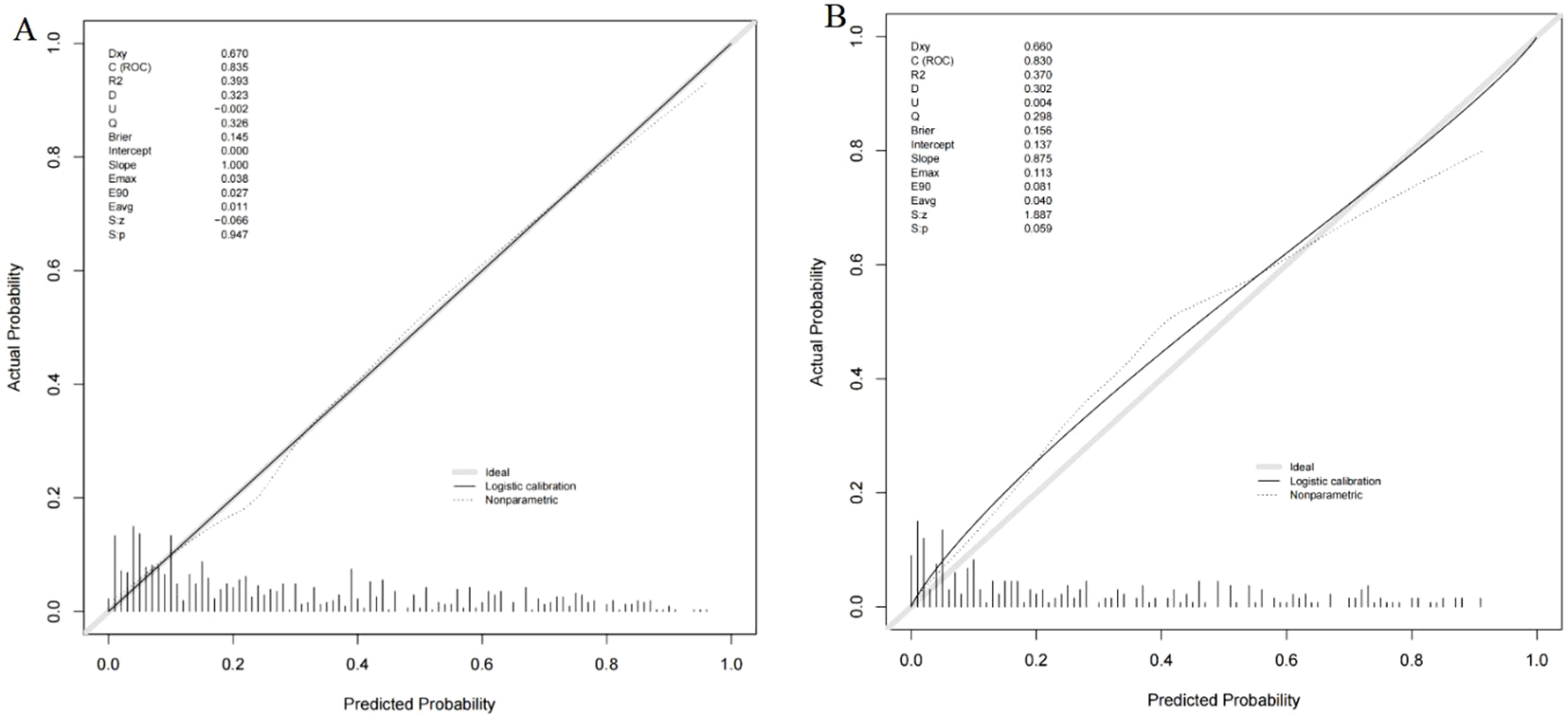
**(A)** Calibration curve of the nomogram in training group. **(B)** Calibration curve of the nomogram in validation group.

**Figure 6:**
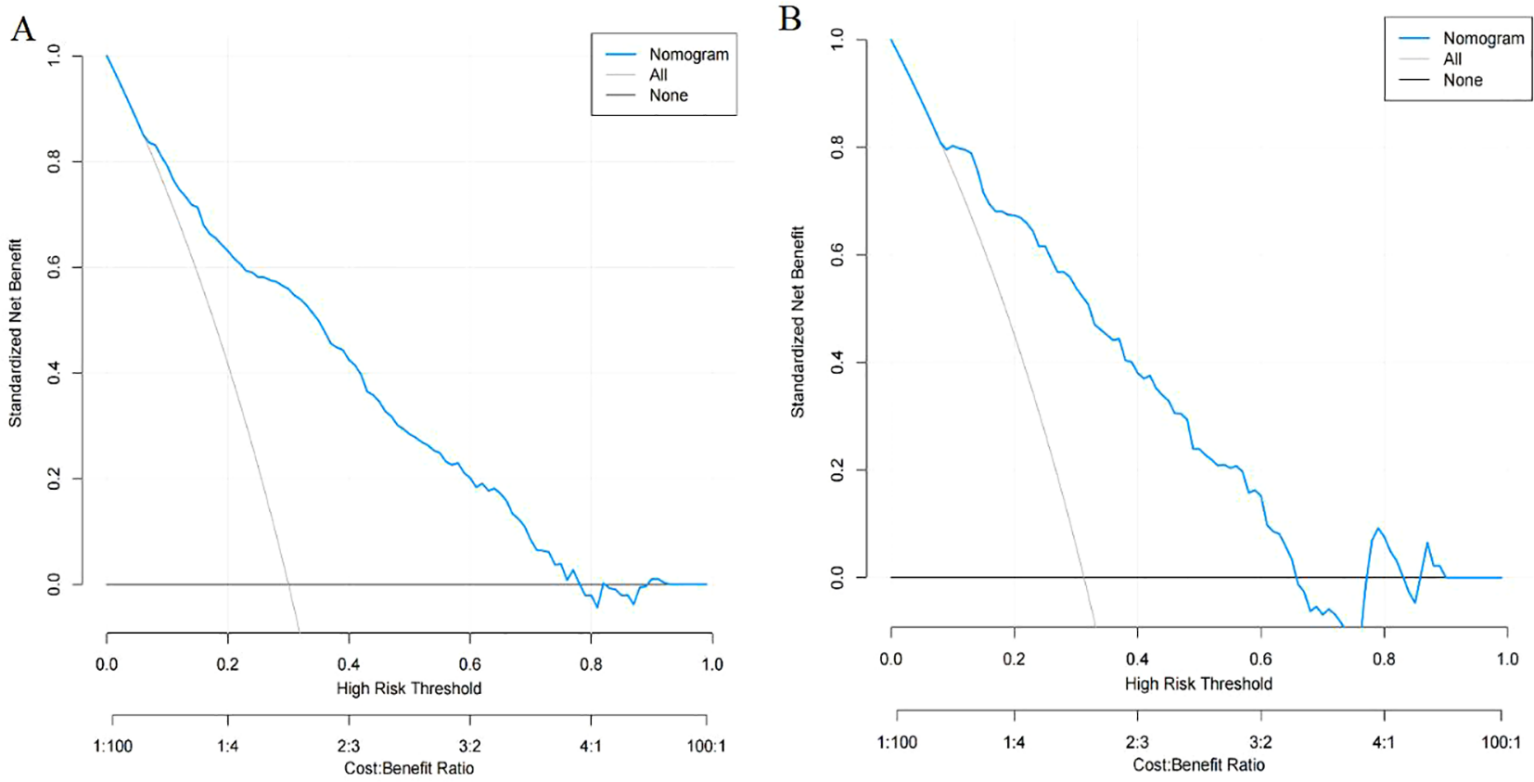
**(A)** Decision curve analysis of the nomogram in training group. **(B)** Decision curve analysis of the nomogram in validation group.

**Figure 7:**
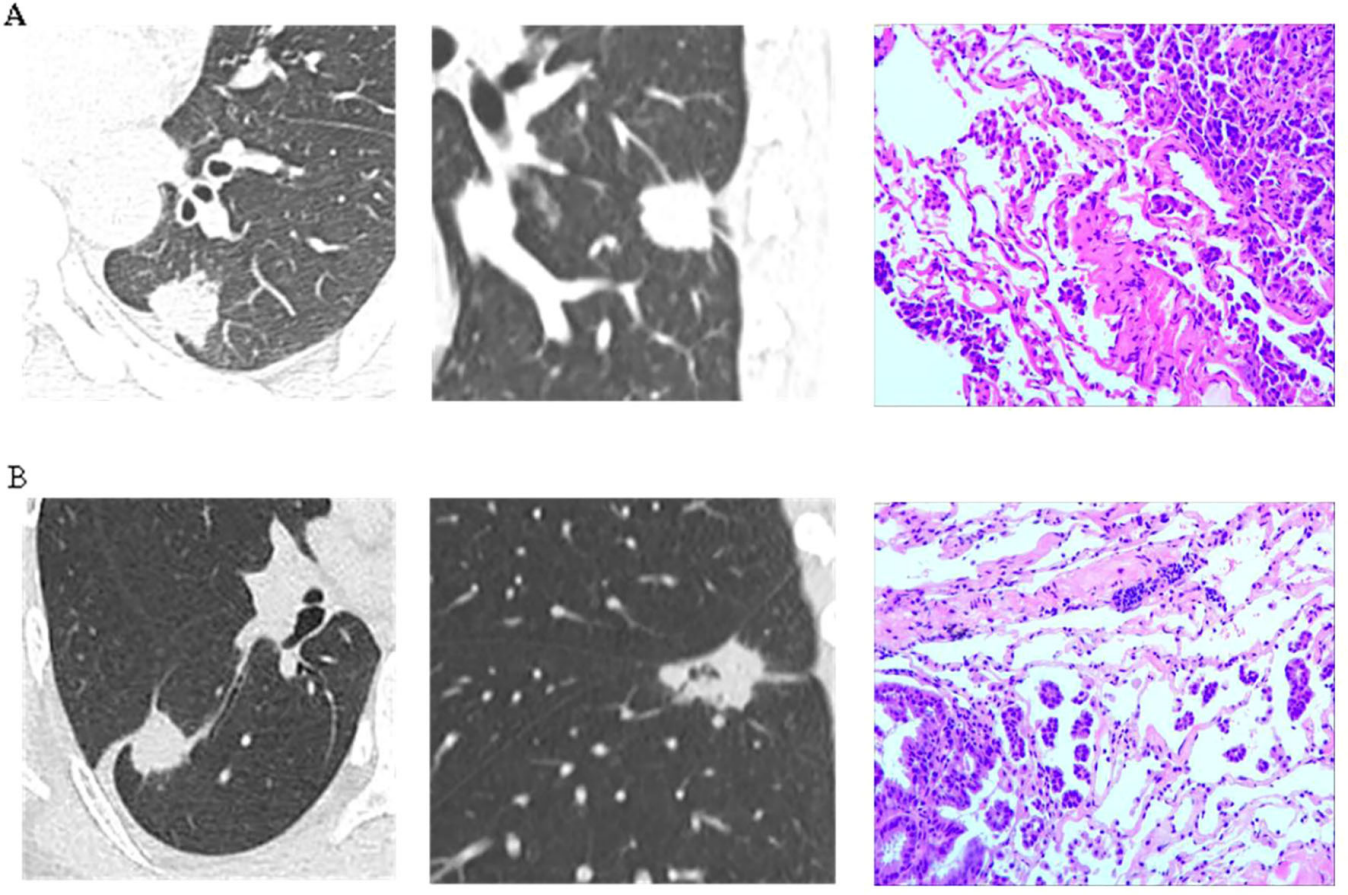
**(A)** A patient was diagnosed with a lesion in the left lower lobe of the lung. Preoperative imaging revealed a solid nodule measuring 2.4 cm in diameter, accompanied by pleural retraction sign and vascular convergence sign. The serum CEA level was 3.0 μg/L. Postoperative pathology confirmed invasive adenocarcinoma with evidence of STAS. **(B)** A patient was found to have a lesion in the right lower lobe of the lung. Preoperative imaging revealed a solid nodule measuring 2.7 cm in diameter, accompanied by pleural retraction sign and vascular convergence sign. The serum CEA level was 4.0 μg/L. Postoperative pathology confirmed invasive adenocarcinoma with evidence of STAS.

A nomogram was constructed using variables such as nodule type, pleural traction, vascular convergence, CEA levels, and maximum diameter to predict STAS in patients with stage IA lung adenocarcinoma (**Figure 2**). The AUC values recorded were 0.835 (95% CI: 0.808–0.862) for the training group and 0.830 (95% CI: 0.782–0.878) for the validation group, indicating a strong ability of the model to differentiate between outcomes in both datasets (**Figures 3**, **4**). The Hosmer-Lemeshow test demonstrated a favorable fit for both the training and validation cohorts (P > 0.05), while the calibration curves illustrated a robust alignment between predicted and observed outcomes, thereby validating the model’s accuracy (**Figure 5**). DCAs for both cohorts highlighted the practical utility of the model (**Figure 6**). Additionally, an AUC of 0.846 (95% CI: 0.818–0.881) obtained through Bootstrap internal validation further affirmed the model’s consistent discriminatory capacity. Application of this model in the validation group demonstrated its robust predictive performance (**Figure 7**).

## Discussion

Lung cancer continues to be a leading factor in cancer-related deaths, with adenocarcinoma identified as the most common subtype (28). The evolution of screening technologies has significantly improved the identification of early-stage lung cancer, positioning surgical intervention as the most effective treatment option for these instances. In light of the findings from the JCOG studies (29–32), sublobectomy has emerged as a prevalent surgical method. Nevertheless, the occurrence of STAS in patients is intricately associated with heightened recurrence rates and reduced survival rates. Lobectomy generally results in more favorable outcomes for individuals diagnosed with STAS-positive stage IA lung adenocarcinoma (11, 33). Therefore, precise preoperative assessment of STAS is crucial for guiding the selection of surgical interventions (8, 10). This study introduced a nomogram that integrates preoperative clinical and CT imaging data to estimate the likelihood of STAS, thereby facilitating surgical planning for early-stage lung adenocarcinoma.

Previous findings by Onozato et al. (15) and Shiono et al. (14) indicate that smoking increases the likelihood of developing STAS, while Warth et al. (17) observed a higher propensity for STAS among male patients, a result that contrasts with ours. In our analysis, although gender and smoking history were significant in univariate analysis, they did not emerge as independent predictors of STAS, aligning with other research (8, 16). This variation could be attributed to differences in the characteristics of the study populations, measurement protocols, and analytical models used in different studies.

Serum tumor markers serve as crucial non-invasive tools for detecting cancer, contributing to early lung cancer screening and monitoring for postoperative progression and metastasis (34). Shimomura et al. (35) reported elevated preoperative CEA levels in STAS-positive patients, a finding echoed by other research (36, 37). Our analysis also identifies high CEA levels as an independent risk factor for STAS. However, Qin et al. (38) did not observe a correlation between CEA levels and STAS, possibly due to the inclusion of patients at various stages of lung cancer.

The role of maximum tumor diameter as a predictor of STAS has been debated. While Toyokawa et al. (39) and others (18) suggest that diameters larger than 2 cm are unreliable predictors, conflicting evidence exists (19, 40). Yin et al. (20) conducted a meta-analysis and found no predictive value for diameters exceeding 2 cm; however, their analysis might have been impacted by treating nodule diameter as a binary variable. In a contrasting study, Qin et al. (38) treated maximum diameter as a continuous variable in a cohort of 503 patients and found it to be an independent predictor of STAS, which supports our conclusions. Our research corroborates that maximum tumor diameter is an independent indicator of STAS in stage IA lung adenocarcinoma, particularly when the diameter surpasses 1.45 cm.

The aggressiveness of lung cancer is closely linked to the proportion of solid tumor components observed on CT scans; a higher solid component indicates a more significant pathological invasion and an elevated probability of STAS. Research by multiple authors (14, 38) has shown a positive correlation between the core-to-total ratio (CTR) and the likelihood of STAS. Qi et al. (41) highlighted CTR as the most accurate CT characteristic for forecasting STAS in lung adenocarcinomas measuring ≤2 cm, whereas Jia et al. (42) demonstrated that an increase in solid components substantially raises the risk of STAS. While several studies (14, 43) have indicated that solid nodules frequently exhibit STAS in imaging, Yin et al. (20) proposed that part-solid nodules might present a greater risk of STAS, potentially reflecting the small number of purely solid nodules in their sample. In their analysis of 327 patients, Toyokawa et al. (19) concluded that a solid component percentage exceeding 50% was predictive of STAS, a finding that contrasts with that of Margerie-Mellon et al. (40), possibly due to variances in nodule size and the heterogeneity of ground-glass components. Our research corroborates that nodule type acts as an independent predictor of STAS, with an increase in solid components markedly heightening the risk.

Qualitative CT features also significantly influence STAS prediction. Yin et al. (20) explored common CT qualitative features linked with STAS, while Gu et al. (21) delved into additional characteristics. Gu’s findings suggest that tumors positive for STAS more frequently exhibited traits such as solid nodules, spiculation, vacuoles, well-defined borders, lobulation, pleural traction, and vascular clustering signs compared to STAS-negative adenocarcinomas. Our findings further substantiate pleural traction signs and vascular convergence as independent risk factors for STAS, with the observed discrepancies potentially arising from our focused analysis on stage IA lung adenocarcinoma patients.

The nomogram prediction model surpasses traditional correlation analyses by synthesizing multiple pertinent features, thereby enhancing both the precision and efficiency of predictions (44, 45). In recent times, radiomics-based nomograms have been devised to improve diagnostic accuracy for STAS. Nonetheless, the practical implementation of these tools is somewhat restricted by the sophisticated technical requirements associated with radiomics (46, 47). Contrastingly, other investigations (23, 37) have employed more straightforward clinical imaging data to estimate STAS risk; however, these studies typically involve smaller cohorts and do not encompass a thorough analysis of both clinical and imaging variables. Variability in study populations further complicates the detailed examination specifically tailored to patients with stage IA lung adenocarcinoma. In our study, we conducted an extensive evaluation of the interplay between clinical and imaging attributes and STAS across a large sample set. We successfully developed and validated a prediction model for STAS in stage IA lung adenocarcinoma, which produced favorable outcomes. When a patient is assessed as high-risk for STAS, the recommended clinical approach includes a lobectomy accompanied by extensive lymph node dissection to optimize prognostic results.

While the findings are promising, it is essential to address several limitations that warrant further examination. The retrospective design of this study presents a challenge in addressing selection bias. The research focuses solely on patients from our institution, classifying it as a single-center retrospective study with a limited sample size. While showing steady performance across different time frames, the lack of multicenter and prospective data restricts the broader applicability and validation of these results. Furthermore, the imaging characteristics were quantified through manual methods, which may introduce a potential for bias.

## Conclusion

By amalgamating clinical and CT imaging features, we crafted a nomogram prediction model capable of forecasting the occurrence of STAS in patients with stage IA lung adenocarcinoma. This model exhibits robust predictive strength for STAS, significantly aiding clinical management and informing decision-making processes concerning this condition.

## Data Availability

The raw data supporting the conclusions of this article will be made available by the authors, without undue reservation.
